# Mildly elevated thyroid-stimulating hormone is associated with endothelial dysfunction and severe preeclampsia among pregnant women with insufficient iodine intake in Eastern Cape province, South Africa

**DOI:** 10.1080/07853890.2021.1947520

**Published:** 2021-07-01

**Authors:** Charles Bitamazire Businge, Benjamin Longo-Mbenza, Andre Pascal Kengne

**Affiliations:** a Department of Obstetrics and Gynaecology, Faculty of Health Sciences, Walter Sisulu University, Mthatha, South Africa; b Department of Medicine, Faculty of Health Sciences, University of Cape Town, Cape Town, South Africa; c Faculty of Medicine, University of Kinshasa, Kinshasa, Democratic Republic of Congo; d LOMO University of Research, Kinshasa, Democratic Republic of Congo; e Non-Communicable Disease Research Unit, South African Medical Research Council, Cape Town, South Africa

**Keywords:** Preeclampsia, iodine deficiency, elevated thyroid-stimulating hormone, pulse wave velocity, endothelial dysfunction

## Abstract

**Background:**

Preeclampsia and hypothyroidism are associated with endothelial dysfunction. Iodine deficiency is a risk factor for subclinical hypothyroidism in pregnancy. However, there is a paucity of data on the relationship between iodine nutrition state in pregnancy, the degree of endothelial dysfunction, and the risk of preeclampsia.

**Methods:**

Ninety-five normotensive pregnant women, 50 women with preeclampsia with no severe features, and 50 women with severe preeclampsia were enrolled into the current study from the maternity units of Nelson Mandela Academic Hospital and Mthatha Regional Hospitals in Eastern Cape Province, South Africa. Urinary iodine concentration (UIC), serum markers of thyroid function, aortic augmentation index, and pulse wave velocity (PWV) were compared.

**Results:**

Median UIC was 167.5, 127.7, and 88.5 µg/L, respectively for normotensive pregnant women, those with preeclampsia and severe preeclampsia (*p* = .150). Participants with severe preeclampsia had significantly higher median thyroid-stimulating hormone (TSH) and oxidized LDL than normotensive and preeclamptic women without severe features (respectively 3.0, 2.3, and 2.3 IU/L; 1.2, 1.0, and 1.0 IU/L, *p* < .05). The median Aortic augmentation index was 7.5, 19.0, and 21.0 (*p* < .001), and the pulse wave velocity 5.1, 5.7, and 6.3, respectively for normotensive, preeclampsia, and severe preeclampsia participants (both *p* < .001). In linear regressions, TSH, age, and hypertensive disease were independent predictors of elevated PWV.

**Conclusion:**

Upper normal-range TSH levels in women with severe preeclampsia were associated with markers of endothelial dysfunction. The low UIC and trend towards the elevation of thyroglobulin suggest that inadequate iodine intake may have increased TSH levels and indirectly caused endothelial dysfunction.

## Introduction

Endothelial dysfunction, which is characterized by atherosis and vasoconstriction, is a precursor of cardiovascular disease [1,2]. Both preeclampsia and hypothyroidism are risk factors for cardiovascular disease among women [3–5]. High serum thyroid-stimulating hormone (TSH) levels above the physiological range, a common feature of subclinical (SCH) and overt hypothyroidism (OH) predispose to endothelial dysfunction through inhibition of endothelial Nitric oxide (NO) synthase [6–8]. This leads to diminished flow-mediated dilation that is modulated by local endothelial NO synthesis in the lumen of the blood vessels [7]. The resultant arterial stiffness is an early marker of incident cardiovascular disease as well as a pathological feature of preeclampsia [1]. Due to the transfer of iodine across the placenta to the growing foetus and the increased physiological renal filtration and loss of iodine in urine during pregnancy, pregnant women in populations with insufficient iodine intake are at increased risk of worsening iodine deficiency [9,10]. This is now thought to lead to subclinical or overt hypothyroidism, endothelial dysfunction, preeclampsia, and other adverse pregnancy outcomes, such as miscarriage, preterm delivery, and intrauterine growth restriction [11–13].

We carried out this case-control study to ascertain how the iodine nutrition status and serum TSH levels varied with the degree of endothelial dysfunction and severity of preeclampsia among women in Eastern Cape South Africa.

## Methods

### Study setting

The participants were enrolled at the maternity units of Nelson Mandela Academic Hospital and Mthatha Regional Hospitals located in OR Tambo municipality one of the rural districts in the Eastern Cape Province, South Africa. South Africa is one of the sub-Saharan countries with iodine-deficient soils [14]. Universal salt iodization has been implemented in South Africa for about 25 years however, 30% of the population especially in rural settings do not have access to adequately iodized salt [14].

#### Sample size calculation

Namugowa and Meeme [15] found the mean pulse wave velocity (PWV) of preeclamptic and normotensive pregnant women of 6.7 ± 1.5 and 5.1 ± 0.7 m/s, respectively (a difference of 1.6 between the two means). Assuming a standard deviation for PWV of 1.5 in our study population, a sample size of 126 (42 preeclamptic and 84 normotensive pregnant women) has the power of 0.80 at an alpha of 0.05 to detect a difference of 0.8 between the mean PWV of preeclamptic and normotensive pregnant women.

### Enrolment of participants

Cases were women diagnosed with preeclampsia according to the criteria stipulated by the International Society for the Study of Hypertension in Pregnancy (ISSHP) [16]. Controls were pregnant women who remained normotensive and presented at term either in latent labour or for delivery by elective caesarean section.

We enrolled 195 participants who voluntarily accepted to participate in the current study (50 women with preeclampsia with no severe features, and 50 women with severe preeclampsia who were matched for chronological age with 95 normotensive pregnant women. The participants with preeclampsia were consecutively enrolled soon after diagnosis while the normotensive pregnant women were selected by simple random sampling of women of similar chronological age who remained normotensive till they presented at term in early labour or for elective caesarean section.

#### Inclusion and exclusion criteria

All women who fulfilled the criteria for cases or controls were eligible for inclusion in the study after providing informed consent. Potential participants who had a history of thyroid disease, those currently with multiple pregnancies, those who had conceived after an artificial reproductive technique procedure, and those with chronic hypertension or a history of diabetes mellitus were excluded.

#### Data collection

Data on demographic and past obstetric history was obtained in addition to the participant's weight and height that were measured using standard procedures. The Blood pressure was measured according to the American Heart Association guidelines, with the patient’s elbow flexed at the heart level. The average of the two measurements with a standard mercury sphygmomanometer taken at intervals ≥2 min after the participants had been sitting for at least 30 min was used [17]. The degree of endothelial dysfunction was determined by calculating the pulse wave velocity and the augmentation index using the SphygmoCor system (version 7.01, Atcor Medical, Sydney, Australia). Venous blood was collected, centrifuged, and the serum aliquoted and stored at −20 °C until analyzed for thyroid function tests (TSH, free thyroxine [FT4], free triiodothyronine [FT3], and thyroglobulin). These were compared with the trimester-specific ranges [18,19]. The TSH, FT4, and FT3 levels were determined using the Roche/Hitachi cobas-c systems, an electrochemiluminescence immunoassay (ECLIA) technology. Mid-stream urine was collected and the Urinary Iodine concentrations (UIC) determined using the inductively coupled plasma (ICP) Mass Spectrometry method according to the manufacturer’s instructions (Quadrupole Inductively Coupled Plasma Mass Spectrometry (X-Series 2 ICP-MS – Thermo-Fisher Scientific, Bremen, Germany) as previously described [20]. Median UIC of <150, 150–249, 250–499, and >500 µg/L, respectively are a measure of insufficient, adequate, more than adequate, and excessive iodine intake during pregnancy [21].

#### Statistical analysis

The IBM SPSS STATISTICS version 22 for windows (IBM Inc., Chicago, IL, USA) software package was used for data analysis. Data were checked to identify variables that were normally distributed using the Shapiro–Wilk’s test. The data was then summarized into proportions (%) for categorical variables, means ± standard deviation (SD) for normally distributed, and as median (p25, p75) for non-normally distributed variables, respectively. The Chi-square test was used to compare the distribution of categorical variables by status for preeclampsia. The Student’s *t*-test, Kruskal–Wallis, and Mann–Whitney *U* tests were used as appropriate for continuous variable comparisons across groups. Univariable and multivariable linear regression was used to investigate the correlates of preeclampsia. A *p*-value <.05 was considered significant.

#### Ethical considerations

The Human Research Ethics Review Committees of Walter Sisulu University and the University of Cape Town approved this study (reference number 066/2017 and 135/2018, respectively). Participation in the study was voluntary with the participants having the freedom to withdraw from the study at any time. All participants that were enrolment in the study provided informed consent.

## Results

The normotensive pregnant women, women with preeclampsia and severe preeclampsia had comparable age (median of 23, 24, and 23 years, respectively) and BMI (median 28, 29.3, and 30.2 kg/m^2^), *p* > .05 (Table 1). The normotensive pregnant women had a higher gestational age at enrolment (median of 39.0 WOA) than women with preeclampsia (34.0 WOA) and severe preeclampsia (32.5 WOA) *p* < .001. The peripheral systolic and diastolic pressure, as well as the aortic systolic and diastolic pressure of normotensive pregnant women, were significantly lower than that of women with preeclampsia and severe preeclampsia *p* < .05, Table 1).

**Table 1.** General characteristics, endothelial, and thyroid function features of cases and controls.

Variable	Normotensive Median (p25, p75)	Preeclampsia Median (p25, p75)	Severe preeclampsia Median (p25, p75)	*p*-Value
Age (years)	23 (20.0, 29.0)	24.0 (20.0, 29.8)	23.0 (20.0, 29.0)	.839
BMI (kg/m^2^)	28.0 (25.9, 31.6)	29.3 (27.0, 38.8)	30.2 (26.2, 34.6)	.180
GA at enrolment (WOA)	39.0 (37.0, 40.0)	34.0 (29.3, 38.8)	32.5 (30.0, 36.3)	<.001
Peripheral systolic BP (mmHg)	121 (114.0, 127)	139.5 (128.3, 145.5)	143.0 (130.8, 158.0)	<.001
Peripheral diastolic BP (mmHg)	76.5 (70.0, 82.0)	90.0 (81.0, 99.0)	95.0 (84.5, 102.3)	<.001
Aortic systolic BP (mmHg)	106.0 (98.5, 115.0)	128.0 (117.0, 138.5)	126.0 (120.0, 144.0)	<.001
Aortic diastolic BP (mmHg)	79.5 (72.0, 84.0)	94.0 (85.8, 94.0)	98.0 (93.0, 124.0)	<.001
Aortic augmentation BP (mmHg)	2.0 (0.0, 5.0)	5.0 (1.0, 10.0)	7.0 (2.0, 13.0)	<.001
Aortic augmentation index	7.5 (-1.0, 18.3.0)	19.0 (6.0, 32.0)	21.0 (8.0, 39.0)	<.001
Pulse wave velocity (m/s)	5.1 (4.7, 5.7)	5.7 (4.9, 6.5)	6.3 (5.7, 7.0)	<.001
OxLDL (IU/L)	1.0 (0.9, 1.2)	1.0 (0.8, 1.2)	1.2 (1.0, 1.3)	.003
TSH (IU/L)	2.3 (1.8, 3.1)	2.3 (1.8, 3.3)	3.0 (2.2, 4.2)	.005
UIC (µg/L)	167.5 (88.5, 295.5)	127.7 (75.8, 364.3)	88.5 (52.6, 550.5)	.150
Thyroglobulin (µg/L)	19.7 (13.0, 34.8)	21.4 (13.2, 36.2)	22.4 (15.0, 38.5)	.785

BMI: body mass index; GA: gestational age; WOA: weeks of amenorrhoea; BP: blood pressure; OxLDL: oxidized low-density lipoprotein; TSH: thyroid-stimulating hormone; UIC: urinary iodine concentration.

The median aortic augmentation pressure was 2.0, 5.0, and 7.0 mmHg, respectively for normotensive, preeclampsia, and severe preeclampsia participants (*p* < .001). A similar pattern was also observed for the Aortic augmentation index (medians of 7.5, 19.0, and 21.0, *p* < .001) and the pulse wave velocity (m/s) (medians of 5.1, 5.7, and 6.3, respectively for normotensive, preeclampsia, and severe preeclampsia participants (*p* < .001, Table 1). Participants with severe preeclampsia had significantly higher TSH and oxidized low-density lipoproteins than the counterparts with preeclampsia or normotensive pregnant controls (*p* < .001, Table 1). There was no significant difference in the urinary iodine concentration (UIC) and thyroglobulin of the three groups (*p* = .785). However, the median UIC of normotensive pregnant women, those with preeclampsia and severe preeclampsia were 167.5, 127.7, and 88.5 µg/L, respectively demonstrating adequate, mild, and moderate insufficient iodine intake in pregnancy that is clinically significant.

We used the second and third-trimester upper serum TSH limit of 4.0 IU/L [18] and the 10th FT4 percentile of 11.3 pmol/L [19] to determine the thyroid function status. The prevalence of subclinical hypothyroidism (SCH) and overt hypothyroidism (OH) were SCH: 8.0, 18.0, and 17.8%; and OH: 1.4, 4.0, and 8.9% for normotensive pregnant women, preeclampsia and severe preeclampsia participants, respectively (Table 2).

**Table 2.** Thyroid function status of normotensive, preeclampsia, and severe preeclampsia participants.

Thyroid status	Normotensive *n* (%)	Preeclampsia *n* (%)	Severe preeclampsia *n* (%)	Chi-square	*p*-Value
Euthyroid	71 (81.6)	34 (68.0)	26 (57.8)	11.601	.07
SCH	7 (8.0)	9 (18.0)	8 (17.8)		
Hypothyroxinaemia	8 (9.2)	5 (10.0)	7 (15.5)		
Overt Hypothyroidism	1 (1.2)	2 (4.0)	4 (8.9)		
Total	87 (100)	50 (100)	45 (100)		

SCH: subclinical hypothyroidism.

Of the three markers of iodine nutrition status (UIC, thyroglobulin, and TSH), TSH was positively correlated with aortic systolic and diastolic pressure and the peripheral diastolic and systolic pressure (Tables 3). UIC was significantly correlated with oxidized LDL (Pearson correlation coefficient 0.205, *p* = .008).

**Table 3.** Non-parametric correlation matrix of iodine nutrition biomarkers and biomarkers of endothelial dysfunction.

	Spearman’s	TSH	UIC	Tg	PWV	ASBp	ADBp	PSBp	PDBp	AABp	AAI
TSH	Rho		−0.079	0.182*	0.113	0.223**	0.255**	0.175*	0.188*	0.058	0.052
	*p*-Value		.319	.022	.147	.004	.001	.023	.015	.456	.502
UIC	Rho	−0.079		−0.042	0.012	−0.093	−0.038	−0.052	0.004	−0.134	−0.131
	*p*-Value	.319		.616	.878	.235	.629	.509	.959	.087	.095
Tg	Rho	0.182*	−0.042		−0.061	0.127	0.082	0.067	−0.031	0.025	0.007
	*p*-Value	.022	.616		.459	.124	.319	.417	.705	.766	.930

TSH: thyroid-stimulating hormone; UIC: urinary iodine concentration; Tg: thyroglobulin; ASBp: aortic systolic blood pressure; ADBp: aortic diastolic blood pressure; PSBp: peripheral systolic blood pressure; PDBp: peripheral diastolic blood pressure; AABp: aortic augmentation blood pressure; AAI: aortic augmentation index.

*p < .05; **p < .01.

Participants with TSH ≥ 4 IU/L, in comparison with those with TSH < 4 IU/L, had higher median pulse wave velocity, and borderline higher aortic systolic and diastolic pressure, as well as peripheral diastolic pressure (5.4 *vs.* 6.0, 115 *vs.* 122, 84 *vs.* 91, and 82 *vs.* 88 mmHg, respectively) (Table 4).

**Table 4.** Median pulse wave velocity, aortic systolic and diastolic pressure, and peripheral diastolic pressure of participants with Thyroid-stimulating hormone levels <4 or ≥ 4 IU/L.

Variables	TS*H* < 4 IU/L Median (p25, p75)	TS*H* ≥ 4 IU/L Median (p25, p75)	*p-Value
Pulse wave velocity (m/s)	5.4 (4.7, 6.3)	6.0 (5.1, 7.1)	.025
Aortic systolic (BP mmHg)	115.0 (104.0, 126.0)	122.0 (105.8, 135.8)	.057
Aortic diastolic (BP mmHg)	84 (77.0, 95.0)	91.0 (81.5, 107.0)	.055
Peripheral diastolic (BP mmHg)	82.0 (75.0, 92.5)	88.0 (77.3, 95.2)	.252

*Mann–Whitney *U* test.

In multivariable linear regression models that included maternal age and hypertensive disease status, two known independent predictors of endothelial dysfunction as well as gestational age, a potential confounder that was statistically significant on univariable analysis, TSH together with maternal age and hypertensive disease status remained independent predictors of PWV (Table 5).

**Table 5.** Linear regression univariable and multivariable beta coefficients of the factors associated with pulse wave velocity.

Variable	Univariable beta coefficient (95% CI)	*p*-Value	Multivariable beta coefficient (95% CI)	*p*-Value
Age (years)	0.036 (0.011–0.061)	.005	0.037 (0.013–0.061)	.003
HDP	0.469 (0.282–0.656)	<.001	0.353 (0.116–0.590)	<.001
TSH (UI/L)	0.142 (0.031–0.253)	.012	0.109 (0.001–0.217)	.048
GA (weeks)	−0.062 (−0.095 to −0.030)	<.001		
Parity	0.106 (−0.05–0.216)	.060		
BMI (Kg/m^2^)	0.008 (−0.20–0.035)	.583		

TSH: thyroid-stimulating hormone; HDP: hypertensive disease in pregnancy; GA: gestational age.

## Discussion

In the current study, the pulse wave velocity (PWV), aortic augmentation index, and augmentation pressure, which are measures of arterial stiffness [22] were significantly increased among women with preeclampsia when compared with normotensive pregnant controls. This is consistent with other studies and implies structural changes in lamina media of the aorta among women with elevated values [23,24]. PWV, an objective measure of endothelial dysfunction and arterial stiffness, becomes elevated secondary to changes in the vascular wall especially a higher collagen-elastin ratio that decreases dispensability [25].

The current study also found that elevated TSH is an independent predictor of PWV after adjusting for maternal age, gestational age, and preeclampsia status. Elevated TSH level, consistent with SCH, was associated with significantly higher median PWV. This is similar to findings by others who reported that even mildly elevated TSH was associated with endothelial dysfunction and structural vascular changes associated with arterial stiffness [26,27] although the study was not conducted among pregnant women. The mildly elevated TSH observed in the current study was not associated with the atherogenic lipid profile that has been reported in some previous studies [28].

Although there was no significant correlation between UIC and TSH, participants with severe preeclampsia had lower median UIC but higher median TSH, which is suggestive of thyroid hyperstimulation possibly due to insufficient iodine intake. This finding correlates with that of Abel et al. [13] who reported that women with insufficient iodine intake at the inception of pregnancy are at increased risk of preeclampsia and other adverse pregnancy outcomes. Screening for iodine deficiency and supplementation is not yet part of routine maternity care in South Africa [29]. Recent iodine intake in pregnancy in South Africa was reported to be adequate [30,31]. However, these surveys did not utilize nationally presentative samples hence could have masked inadequate iodine intake in pregnancy in some local geographical settings.

The degree of iodine deficiency and the levels of oxidized LDL both increased with the severity of preeclampsia. Oxidized LDL is a known precursor of atherosis, endothelial dysfunction, and arterial stiffness [28]. Hence, the observed relationship between PWV and TSH could partially be mediated by the underlying exposure to iodine deficiency causing reduced anti-oxidant status [32] and the resultant increase in oxidized LDL.

Others have found elevated TSH to be associated with increased carotid media thickness, which like PWV, is an early marker of atherosclerosis and future cardiovascular disease [33]. This is mediated through stimulation of endothelial TSH receptors, inhibition of endothelial nitric oxide synthase, NO depletion resulting in reduced flow-mediated dilation and endothelial activation [6–8,33].

The increase in arterial stiffness among women with preeclampsia has been found to persist up to ten years post-delivery and may in part be responsible for the increased risk of future cardiovascular disease and end-stage renal failure [3,34–36]. If the observed relationship in the current study between iodine deficiency and TSH that seems to increase the risk and severity of endothelial dysfunction and preeclampsia is confirmed by future studies, it will be worthwhile to provide supplementation for pregnant women who live in geographical locations and populations with known insufficient iodine intake.

### Strengths and limitations

This study has found a probable association between iodine deficiency-mediated TSH elevation, endothelial dysfunction, and preeclampsia which if confirmed by further research can be prevented with iodine supplementation. However, this study is limited by the indirect measurement of endothelial dysfunction as well as non-measurement of potential confounders of the association between preeclampsia and endothelial dysfunction, such as endothelin. Several women with preeclampsia had spent some days admitted to the hospital where they were exposed to meals prepared with adequately iodized salt that may have significantly affected their UIC. Although median spot UIC is recommended as a measure of iodine nutrition status at the population level, it is not a robust measure of the iodine nutritional status at the individual level [21,37].

## Conclusions

Preeclampsia is characterized by increased PWV, aortic augmentation index, and aortic augmentation pressure, which are markers of arterial stiffness and predictors of future cardiovascular disease. Elevated TSH increases the risk of endothelial dysfunction in pregnancy even when it is within levels suggestive of mild subclinical hypothyroidism as observed in the current study.

## Data Availability

The data set for this study can found at the University of Cape Town Ziva hub repository (doi: 10.25375/uct.14169695).
